# Factors Implicated in Radiation Therapy Failure and Radiosensitization of Prostate Cancer

**DOI:** 10.1155/2012/593241

**Published:** 2011-09-08

**Authors:** Helmut Bonkhoff

**Affiliations:** Pathology Laboratory, Großbeerenstraße 12, 12209 Berlin, Germany

## Abstract

Tissue markers may be helpful in enhancing prediction of radiation therapy (RT) failure of prostate cancer (PCa). Among the various biomarkers tested in Phase III randomized trials conducted by the Radiation Therapy Oncology Group, p16, Ki-67, MDM2, COX-2, and PKA yielded the most robust data in predicting RT failure. Other pathways involved in RT failure are also implicated in the development of castration-resistant PCa, including the hypersensitive androgen receptor, EGFR, VEGF-R, and PI3K/Akt. Most of them are detectable in PCa tissue even at the time of initial diagnosis. Emerging evidence suggests that RT failure of PCa results from a multifactorial and heterogeneous disease process. A number of tissue markers are available to identify patients at high risk to fail RT. Some of these markers have the promise to be targeted by drugs currently available to enhance the efficacy of RT and delay disease progression.

## 1. Introduction

Radiation therapy (RT), including brachytherapy, external beam radiation, and proton therapy, is one of the most popular treatment options for clinically localized prostate cancer. A major problem with external beam radiation of solid tumors is the limited amount of radiation which can be safely delivered to the target organ. For prostate cancer, radiation doses are generally limited to <80 Gray because of the increased risk of toxicity at higher doses and the lack of clinical evidence that doses >80 Gray improve local tumor control. New strategies aim to sensitize tumors to radiation [1]. Androgen deprivation therapy (ADT) in combination with RT has been so far the only successful sensitizing strategy that has improved cause-specific survival in men with locally advanced prostate cancer [2, 3]. 

Various genetic and epigenetic abnormalities have been associated with radiation-resistant PCa (RRPCa). The Radiation Therapy Oncology Group (RTOG) can be credited with having performed the most extensive studies of biomarkers in men with clinically localized prostate cancer treated with external beam radiotherapy on two phase III randomized trials, RTOG 8610 and RTOG 9202. To date, preliminary assessments of p53, DNA ploidy, p16/pRB, Ki-67, MDM2, bcl-2/bax, and CAG repeats, COX-2, Stat3, Cyp3A4, and protein kinase A (PKA) have been completed [4]. It is noteworthy that most of these tissue markers are not specific for RT failure and may have also prognostic implications in PCa patients treated with surgery and androgen deprivation.

The present review is focused on pathways implicated in RT failure of PCa to define etiological factors which can be targeted by drugs currently available to enhance the efficacy of RT and delay disease progression.

## 2. Anatomic Factors Implicated in Radiotherapy Failure 

PCa cells located at the limit of and outside the radiation field will fail RT. Perineural invasion is a common pathway by which PCa cells transverse the prostate capsule to reach the extraprostatic tissue. Clinical studies have identified perineural invasion present in prostate biopsy an independent risk factor for recurrence and cause-specific death after RT [5, 6]. 

**Figure 1:**
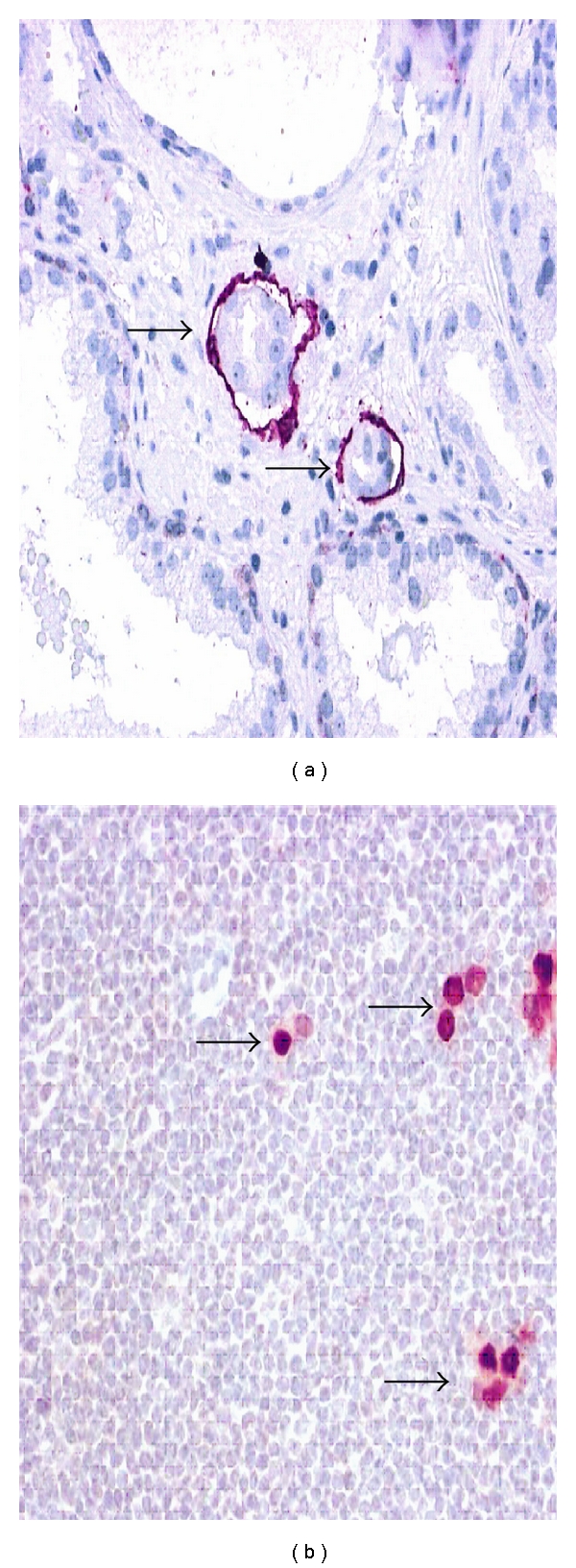
Lymphovascular invasion is detected by the endothelial marker D2-40 in a prostatectomy specimen (a). Androgen receptor (AR) immunohistochemistry detects disseminated prostate cancer cells in lymph nodes classified as negative for cancer upon routine histological examination. Original magnifications: (a) (×400), (b) (×300).

Lymphatic invasion is the first step to lymphatic spread and lymph node metastasis (Figure 1(a)). In prostatectomy specimens, the presence of lymphatic invasion detected by the endothelial marker D2-40 is a major risk factor of lymph node metastasis at the time of prostatectomy, an independent risk factor for PSA recurrence and cancer death in patients treated with prostatectomy, and an independent risk factor for disease recurrence after salvage RT [7–11]. 

Disseminated tumor cells (occult metastases) are detectable by immunohistochemical markers (keratins, PSA, androgen receptor) in lymph nodes qualified as negative (pN0) upon histological examination (Figure 1(b)). In a recent study of 180 patients with pathological stage pT3, pN0, occult lymph node metastases (OLN+) were found in 13.3%. The presence of OLN+ was significantly associated with increased recurrence and decreased survival compared with OLN negative patients (*P* < 0.001 and *P* = 0.019, resp., relative risk of recurrence, 2.27; relative risk of death 2.07, resp.). The presence of occult lymph node metastases was an independent predictor of recurrence and death in a multivariable analysis. The outcome for patients with OLN+ disease was similar to that for patients with histological evidence of lymph node metastases (pN1) [12]. Hence, the presence of disseminated tumor cells (occult metastases) in lymph nodes qualified as negative (pN0) is not a rare event in pT3, pN0 disease, and is an important risk factor of therapy failure after salvage RT.

It is noteworthy that neither lymphatic invasion (pL1) nor disseminated tumor cells in lymph nodes (OLN+) are considered in current nomograms, and thus escape clinical risk evaluation.

## 3. Pathways Implicated in Radiotherapy Failure 

Major pathways implicated in RT failure in solid tumors refer to tumor cell proliferation, resistance to apoptotic cell death, aberrant growth factor receptor expression, and hypoxia. In PCa, the first three pathways are closely entangled to androgen receptor (AR) signaling, which plays a pivotal role in progression to castration-resistant PCa (CRPCa). In this review we adapt current models proposed for the pathogenesis of CRPCa [13–16] to discuss basic mechanisms involved in RRPCa. Current pathogenetic concepts implicate CRPCa a multifactorial and heterogeneous disease process involving several pathways [13–23] including the following.

- Upregulation of AR expression in PCa cells maintaining AR signaling under standard ADT (hypersensitive pathway).
- Enhanced ligand-dependent activation of the AR by increase of intratumoral de novo synthesis of testosterone and dihydrotestosterone (DHT).
- Ligand-independent activation of the AR by non-hormonal growth factor receptors (erb1/EGFR, erb2/HER2, etc.; outlaw pathway).
- Broadened ligand specificity of AR mutants binding nonandrogen steroids (estrogens, progestins, etc.; promiscuous pathway).
- AR-independent mechanisms (bcl-2, neuroendocrine (NE) differentiation, etc.) maintaining survival and growth by bypassing the AR (bypass pathway).
- Continuous resupply of tumor cell populations under ADT by prostate cancer stem cell regeneration (stem cell pathway).

As it will be discussed in the following sections, most of these pathways (a, b, c, e, and f) are also relevant for the development of RRPCa. AR mutations (d) mainly occur in metastatic lesions and are unlikely to play a major role in RT failure of PCa.

### 3.1. Hypersensitive Pathway

**Figure 2:**
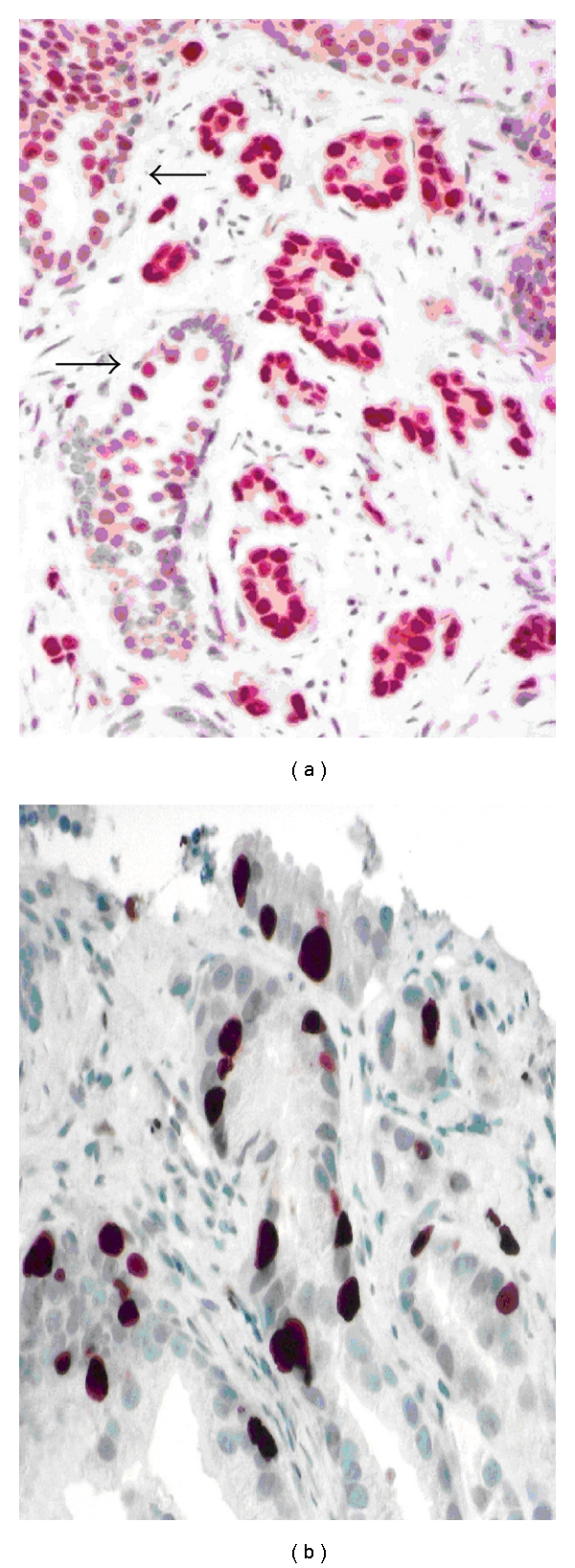
Androgen receptor (AR) status in hormone- and radio- naïve PCa, Gleason 3 + 4 = 7 (Figure 2(a)). The AR is expressed at higher levels when compared with adjacent benign acini (arrows), indicating a hypersensitive pathway. Proliferation activity usually correlates with the Gleason grade. Figure 2(b) shows an example of a PCa, Gleason 3 + 3 = 6, with relatively high proliferation activity (MIB-1 index > 10%), which implies a higher risk of RT failure than the Gleason grade would suggest. Original magnifications: (a) (×300), (b) (×400).

The AR governs vital functions of PCa cells, such as proliferation and survival, and is therefore involved in the biology of RRPCa. Experimental data have convincingly demonstrated that upregulation of the AR at the mRNA and protein level is sufficient to convert castration-sensitive to castration-resistant PCa growth. This hypersensitive pathway has been recognized as a major determinant for the development of CRPCa by maintaining AR signaling during standard ADT [19]. High level of AR expression has been documented by immunohistochemistry not only in CRPCa, but also in hormone-naïve PCa (Figure 2(a)). Several clinical studies performed in hormone-naïve PCa obtained from prostatectomy specimens have shown that high levels of AR expression correlate with the Gleason grade, pathological stage, lymph node status, and PSA recurrence, suggesting that PCa expressing the AR at high levels behave clinically more aggressively than tumors without AR upregulation [24–28]. Since ADT in combination with RT has been demonstrated to improve cause-specific survival [2, 3], it is likely that the AR status in PCa tissue may predict the outcome of patients treated with radiation. Recent data indicate that increased AR expression in PCa detected in biopsy specimens significantly predicts resistance to therapy, that is ADT with or without salvage radiotherapy, and any clinical failure [28]. Nevertheless, further studies are required to determine the significance of the AR status in PCa tissue for predicting RT failure.

Unopposed tumor cells proliferation under ADT is one of the hallmarks of CRPCa, but is also relevant in the development of RRPCa. In the RTOG 8610 and the subsequent RTOG 9202 trail, the proliferation-associated Ki-67 (MIB-1) labeling index ≤3.5% and >3.5%, was associated with the risk of distant metastasis (*P* < 0.0001), disease specific survival (*P* < 0.0001), and overall survival (*P* < 0.01), and was the most significant predictor of the first two endpoints [4] (Figure 2(b)). It is not surprising that some of the key regulators of the cell cycle in PCa cells (i.e., PTEN (phosphatase and tensin homologue), p27, and p16) are implicated in RT failure. When PTEN is lost on chromosome 10 (which occurs in more than 20% of PCa with Gleason ≥7), the phosphatidylinositol 3 kinase (PI3K)/AKT/mammalian target of rapamycin (mTOR) pathway is activated leading to phosphorylation of the AR (→ hypersensitive AR), and increase of cell proliferation by downregulating p27 [17]. In fact, p27 prevents PCa cells from entry into the cell cycle and functions as tumor suppressor. Loss of p27 in PCa demonstrated by immunohistochemistry has prognostic implications (reviewed by Quinn et al. [29]). In castration-sensitive PCa, ADT increases p27 expression which, in turn, prevents PCa cells from proliferation. In CRPCa, p27 is lost, implicating that tumor cells continue to proliferate under ADT. Low expression of p27 in salvage prostatectomy specimens have been reported a significant predictor of survival for patients with recurrent disease after RT [30]. In the radioresistant PCa cell lines PC-3 and DU-145, PTEN is deleted and the PI3K/AKT pathway suppressed. Restoration of PTEN sensitizes these cells to the killing effect of radiation [1]. 

p16 is another important regulator of the cell cycle at the G(1) phase. In the RTOG 8610 trail, loss of p16 expression was associated with an increased risk of local failure, distant metastasis, and disease-specific survival (*P* < 0.01; *P* < 0.03; and *P* < 0.01, resp.) and there was a borderline association with overall survival (*P* = 0.07) [4]. In the subsequent RTOG 9202 study enrolling 612 patients, reduced expression was associated with an increased rate of distant metastases (*P* < 0.04). Among patients with high expression of p16, the use of long-term ADT was associated with an increase in cause-specific survival and a decreased incidence of distant metastasis compared to short-term ADT, suggesting that patients with high p16 expression require long term ADT [4].

The protein kinase A type 1 (PKARI*α*)) belongs to a family of cyclic AMP-dependent enzymes that are involved in the hypersensitive pathway by regulating both the level of AR expression and cell proliferation. Preclinical data indicate that PKARI*α* knockdown with antisense oligonucleotides significantly inhibits PCa cell growth in vitro and in vivo when combined with ADT and RT, and downregulates expression of the AR (AR silencing) [31]. In the RTOG 8610 and the subsequent RTOG 9202 trail, staining intensities were independent predictors of distant metastasis (*P* < 0.01), local failure (*P* < 0.05), and biochemical failure (*P* ≤ 0.01). Furthermore, the benefit of long-term ADT over short-term ADT was much less when PKARI*α* expression was high [32]. 

Cumulatively, the current data suggest that high proliferation activity associated with deregulated cell cycle control (hypersensitive AR, PTEN/PI3K/Akt, p16, and PKA) are involved in RT failure in PCa.

### 3.2. Outlaw Pathway

PCa cells can maintain AR signaling, proliferation, and survival in an androgen-deprived milieu through activation of nonsteroidal growth factor receptors, including vascular endothelial growth factor receptor (VEGFR), insulin-like growth factor receptor (IGFR), keratinocyte growth factor receptor (KGFR), epidermal growth factor receptor (EGFR), transforming growth factor receptor *β* (TGF *β*-R), and interleukine-6 receptor (IL6-R). Phosphorylation of the AR by growth factors and their receptors requires mitogen-activated protein kinases (MAPK) and PI3K/AKT/mTOR downstream kinase signaling [13–18, 20, 22]. These nonsteroidal receptor-related outlaw pathways not only maintain and increase AR signaling (→ hypersensitive pathway), but are also involved in RT failure through various mechanisms. 

**Figure 3:**
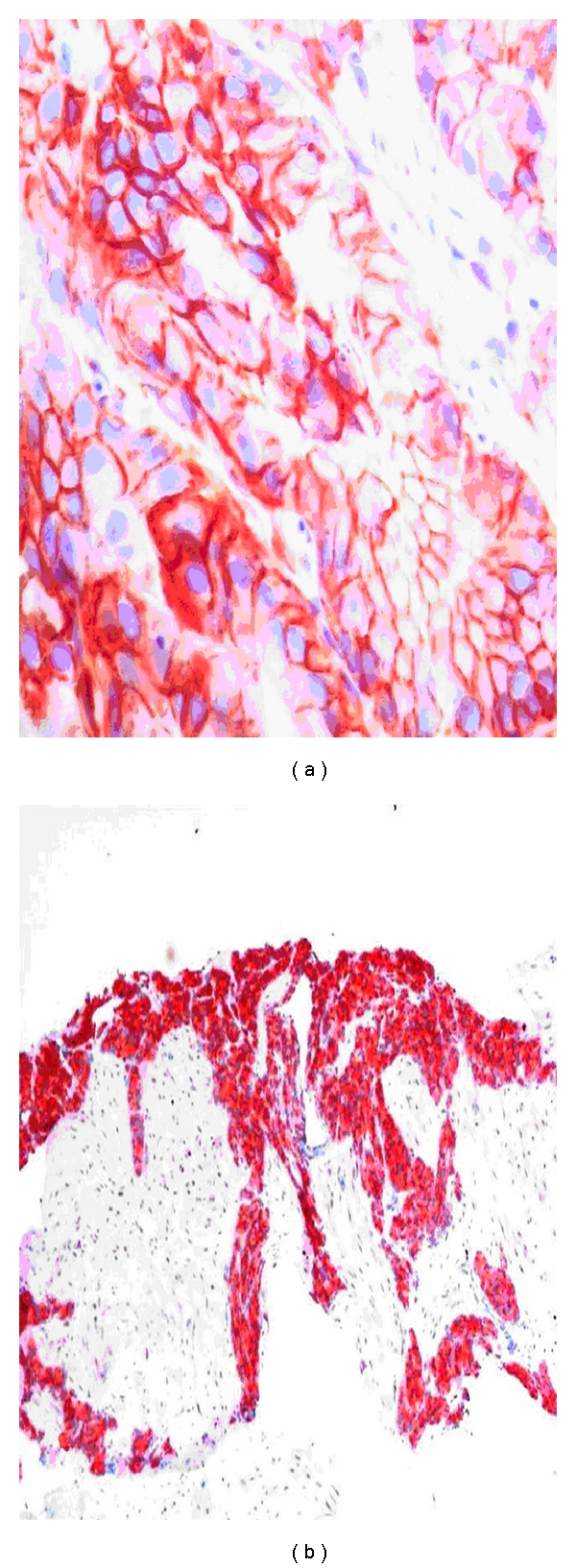
Hormone- and radio-naïve PCa (Gleason 4 + 4 = 8) with strong membraneous HER2/neu expression (Figure 3(a)), indicating that the outlaw pathway is relevant for tumor progression under standard ADT and RT. Local PCa recurrence after salvage radiotherapy (Figure 3(b)). High levels of bcl-2 expression indicate that standard ADT is insufficient for local tumor control. Since docetaxel inhibits the antiapoptotic function of bcl-2, ADT combined with docetaxel may be more successful for local tumor control than standard ADT alone. Original magnifications: (a) (×400), (b) (×100).

Activation of EGFR family members (erb1/EGFR, erb2/HER2, erb3/HER3, and erb4/HER4) via the PI3K/Akt/mTOR pathway is implicated in radioresistance by regulating hypoxia-inducible factor 1*α* (HIF-1*α*) and vascular endothelial growth factor (VEGF) expression, a potent mediator of angiogenesis (reviewed by Karar and Maity, [33]). In addition, radiation can enhance EGFR expression, which, in turn, increases radioresistance of cancer cells. In PCa, upregulation of EGFRs (such as erb1/EGFR, erb2/HER2) are mainly encountered in tumors with high Gleason grades and predict poor outcome [29] (Figure 3(a)). Detection of EGFRs at high levels in the patient's PCa tissue may be a significant risk factor of RT failure, but this has not yet been investigated. 

Hypoxia and acidification caused by increased glycolysis and CO2 production are other significant determinants in RT failure (reviewed by Karar and Maity, [33]). The acidic and hypoxic microenvironment can make cells resistant to both radiation and chemotherapy. Hypoxic cells require higher doses of radiation compared to oxic cells to achieve the same cytotoxic effect. In fact, oxygen present during radiation exposure is critical to elicit maximal DNA damage through the creation of free radicals [33]. The hypoxic microenvironment upregulates not only the expression of HIF-1 and VEGF (via the PI3K/Akt/mTOR pathway), but also induce the transcription factors nuclear factor-kappa B (NF-*κ*B) and signal transducers and activators of transcription 3 (Stat3), which are constitutively active in most cancers, including PCa. NF-*κ*B regulates AR expression and various other pathways implicating in inflammation (IL-6, IL-8, COX-2), cell survival (BCL-2), cell proliferation (cyclin D1, c-myc), invasion, angiogenesis (VEGF, FGF), and metastasis (reviewed by Aggarwal et al., [34]). Increased *NF-*κ*B* expression levels are associated with PCa progression and the development towards castration-resistant disease [35, 36]. Interestingly, *NF-*κ*B is regulated by the PTEN/*PI3K/AKT pathway. The significance of *NF-*κ*B expression *in human PCa tissue for prediction of RT failure has not yet been investigated.

Recent data suggest that Stat3* is involved* in prostate cancer metastases. Stat3 is constitutively active in 77% of lymph node and 67% of bone metastases of clinical PCa [37]. Stat3 expression was evaluated in a subset of 62 patients who had sufficient tissue from RTOG 8610 [4]. Activated Stat3 was inversely correlated with the development of distant metastasis (*P* = 0.04), but not survival or local control, although due to the small sample size this conclusion has to be interpreted with caution [4].

In summary, a number of biomarkers implicated in the outlaw pathway of CRPCa are potential targets for radiosensitization, including erb1/EGFR, erb2/HER2, erb3/HER3, *PTEN*/PI3K/AKT, HIF-1, VEGF-R, *NF-*κ*B,* and Stat3.

### 3.3. Bypass Pathway

Several mechanisms have been recognized of how PCa cells survive ADT by bypassing the AR. The bypass pathway mainly refers to the programmed cell death, which is a major molecular determinant of radioresponse.

**Figure 4:**
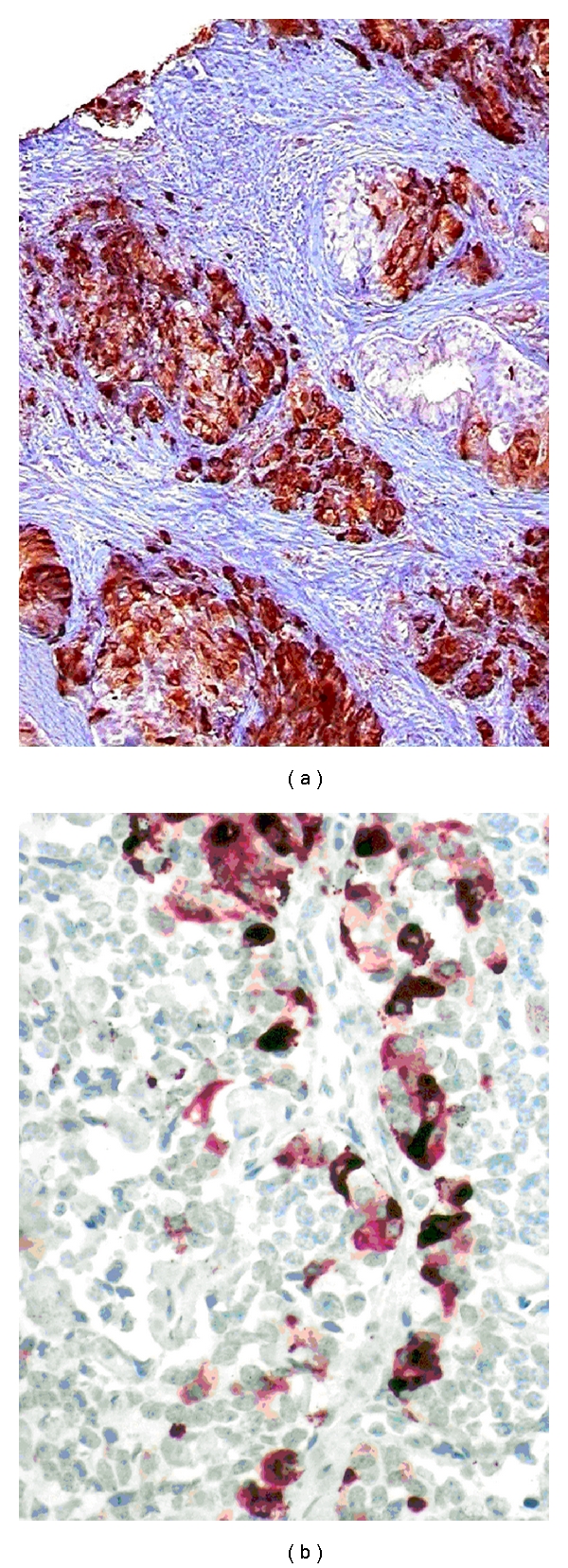
Conventional PCa (Gleason 4 + 4 = 8) detected in a needle biopsy. Chromogranin A (CGA) immunohistochemistry reveals extensive neuroendocrine (NE) differentiation, which presents a multidrug-resistant phenotype in PCa (a). Immunohistochemical detection of somatostatin receptors in NE PCa cells (b) provides a therapeutic target for somatostatin analogues. Original magnifications: (a) (×100), (b) (×300).

*Bcl-2 *is a mitochondrial protein conferring resistance to programmed cell death. The basal cell layer which harbors the stem cell and proliferation compartment of the prostatic epithelium uniformly expresses bcl-2 conferring basal cells resistance to various apoptotic conditions. In fact, basal cells are particularly resistant to radiation and other cytotoxic agents such as ADT and chemotherapy. In PCa, overexpression of bcl-2 is related to high grades and advanced stages, and is considered a prognostic marker for poor outcome and resistance to ADT (reviewed by Quinn et al. [29] (Figure 3(b)). Bcl-2 expression may also be induced or increased by ADT and RT (Figure 4(b)). In a study including 20 radio-naïve and 20 radio-recurrent PCa, all radio-naïve tumors were bcl-2 negative while 55% of the radio-recurrent tumors were bcl-2 immunopositive [38]. Bcl-2 and the proapoptotic bcl-2 family member Bax were evaluated in the RTOG 8610 study, but both were not related to outcomes. In the follow-up RTOG 9202 study, the combination of negative bcl-2/normal bax expression was related to reduced biochemical failure (*P* = 0.036), particularly among those who received short-term ADT suggesting that long-term ADT might be advised when either bcl-2 or Bax is abnormally expressed [4].

Neuroendocrine (NE) differentiation frequently occurs in common PCa but usually escapes histological and clinical detection [39–41]. NE tumor cells do not secrete PSA and become detectable in PCa tissue only upon immunohistochemical analysis. About 10% of PCa (mainly poorly differentiated tumors) show significant (extensive and multifocal) NE differentiation with the NE marker chromogranin A (CGA) [39] (Figure 4(a)). NE PCa cells consistently lack the AR and present an androgen-insensitive phenotype in all stages of the disease [40, 41]. NE differentiation exclusively occurs in the G0-phase of the cell cycle, in which tumor cells are particularly resistant towards cytotoxic conditions such as RT and chemotherapy [40, 41]. Although NE PCa cells do not proliferate, they produce a number of NE growth factors, including serotonin and bombesin that trigger cell proliferation of adjacent exocrine tumor cells through a paracrine mechanism [40, 41]. They also regulate angiogenesis by secreting VEGF. Most strikingly, NE PCa cells escape programmed cell death and represent a potential immortal tumor cell population in PCa [42]. The stem cell marker CD44 is expressed in PCa selectively in NE tumor cells, indicating that these cells are endowed with stem cell properties [43]. Given its multidrug-resistant nature, it is not surprising that NE differentiation may significantly increase under ADT and RT. Pretreatment-elevated serum NSE (neuron-specific enolase), but not initial CgA, has been associated with an unfavorable prognosis in patients treated with RT [44, 45]. In short, NE differentiation characterizes a multidrug-resistant phenotype in common PCa, and may be significant in at least 10% of PCa patients. Unfortunately, NE differentiation usually escapes clinical detection and attention.

*COX-2 *(cyclooxygenase-2) is a proinflammatory enzyme that converts arachidonic acid to prostaglandins, particularly PGE2. Inflammatory processes are implicated in PCa development and tumor progression. During tumor progression, the androgen-sensitive prostatic stroma is replaced by an androgen-insensitive tumor stroma, in which factors such as hypoxia, acidosis and inflammation promote the release of growth factors implicated in outlaw pathways, and upregulation of COX-2 in PCa cells [14, 34]. In these inflammatory tumor-microenvironment interactions, COX-2 and PGE2 play a key role. Inhibition of the COX-2 pathway downregulates a number of important targets of CRPCa, including AR, EGF-R, AKT, and cyclin D [46]. Other preclinical data have shown that COX-2 overexpression and subsequently targeting COX-2 with COX-2 inhibitors may render cells susceptible to the killing effects of radiation (reviewed by Rosser et al. [1]). Similar to what was reported for bcl-2, cancer cells exposed to low doses of radiation up-regulate COX-2 expression as a possible means to survive the radiation exposure [1]. Results of the RTOG 9202 study have identified COX-2 an independent marker for therapy failure after external radiation. In multivariate analyses, the intensity of COX-2 staining was an independent predictor of distant metastasis, biochemical failure, and any failure [47]. Hence, COX-2 offers a relevant therapeutic target in PCa's patients treated with radiation, provided that COX-2 is present in the patient's PCa tissue.

p53 functions by regulating the transcription of genes involved in G1-phase growth arrest and apoptosis in response to DNA damage. Frequently mutated and upregulated in high-grade and metastatic disease, abnormal accumulation of p53 in PCa tissue have been associated with PCa progression and poor outcomes [29]. In the RTOG 8610 and 9202 studies, abnormal p53 expression was significantly associated with risk of distant metastasis and cause-specific survival [4]. 

MDM2 acts as an oncoprotein promoting p53 degradation. A recent study has combined MDM2 with MIB-1 (Ki-67) in 478 patients treated on RTOG 9202. The results indicate that combined MDM2 and MIB-1 expression levels are independently related to distant metastasis and mortality, and is promising in identifying men at a particularly high risk of distant metastases [48]. Experimental data indicate that antisense-MDM2 sensitizes LNCaP prostate cancer cells to androgen deprivation and radiation [49].

### 3.4. Stem Cell Pathway

Some pathogenetic factors implicated in progression towards CRPCa and RRPCa are also required for survival and growth of the normal prostatic epithelium exposed to ADT, RT, and other cytotoxic conditions. The cellular integrity of the prostatic epithelium is maintained by basal cells which are particularly resistant to ADT, chemotherapy, and radiation. Basal cells, in apparent contrast to secretory luminal cells, are endowed with multidrug resistance properties. The basal cell layer of the prostatic epithelium is androgen-independent and harbors the proliferation and the stem cell compartment of the prostatic epithelium [50]. Bcl-2, involved in bypass pathways of CRPCa, and RRPCa, is the major antiapoptotic protein protecting basal cells from programmed cell death. Non-hormonal growth factor receptors (erb1/EGFR, erb2/HER2, etc.) responsible for outlaw pathways implicated in CRPCa and RRPCa are selectively expressed in the basal cell layer [50]. Hence, basal cells, RRPCa and CRPCa cells share common multidrug resistance pathways. The progressive emergence of basal cell specific pathways (including bcl-2, erb1/EGFR, erb2/HER2) during progression towards CRPCa and RRPCa phenotypes suggests that these tumors recapitulate biological properties of basal cells and stem cells to acquire multidrug resistance. 

The basal cell layer of the prostatic epithelium harbors a small stem cell population (<1%) which expresses CD44, *α*2*β*1 integrin and CD133 [51]. The same marker profile has been identified in about 0.1% of tumor cells of any PCa. These very rare tumor cells possess a significant capacity for self-renewal and most likely represent prostate cancer stem cells [52]. PCa stem cells are considered multidrug-resistant tumor cells that continually resupply tumor cells under ADT, RT and others cytotoxic conditions, and may be responsible for therapy failure. It has been shown recently that most lethal metastatic PCa arise from a single precursor cancer cell, and that PCa stem cells are able to generate highly tumorigenic cell populations [51]. Detection of putative stem cell markers in PCa specimens by immunohistochemistry may become important in the near future to select patients for targeting the stem cell pathway.

## 4. Potential Therapeutic Targets for Radiosensitization of Prostate Cancer

Disease relapse after definitive RT is usually identified by elevated or rising serum PSA profiles during and after treatment. Nevertheless, relevant pathogenetic factors implicated in the development of RRPCa are detectable in radio-naïve PCa long before the clinical onset of the disease. This includes pathways related to AR, PTEN/PI3K/AKT, EGFR's, VEGFR, NF-*κ*B/stat3, bcl-2, COX-2, and CGA. Although these pathways and related tissue markers are not specific for radioresistance, RT may induce and enhance relevant pathogenetic factors involved in RT failure, including upregulation of HER1, bcl-2, CGA, COX-2, and NF-*κ*B/stat3. 

The following predictive markers are promising to identify PCa patients at high risk to fail RT and to provide therapeutic targets for radiosensitization to slow disease progression (Table 1).

### 4.1. Targeting Disseminated Tumor Cells

Detection of peritumoral lymphatic invasion (pL1) and occult lymph node metastasis (OLN+) by immunohistochemistry in lymph nodes reported as negative for cancer are significant risk factors for tumor recurrence after salvage radiation therapy [11, 12]. In patients with PSA recurrence after prostatectomy and clear evidence of pL1 or OLN+, extension of the radiation field to the pelvic lymphatic drainage may improve clinical outcome (Figure 1).

### 4.2. Targeting the Hypersensitive Pathway

Determination of the AR status by immunohistochemistry provides a powerful tool for predicting therapy failure after prostatectomy and response to ADT with or without salvage radiotherapy [28]. Detection of high level expression of AR in hormone- and radio-naïve PCa cells implicates the hypersensitive pathway as relevant for the individual disease process (Figure 2(a)). In this case, a standard ADT is not sufficient, because hypersensitive ARs use androgens at castration levels.

AR silencing by downregulating AR expression is a new approach for targeting the hypersensitive pathway [14, 17, 21, 22]. Experimental data using synthetic small interference RNA and AR antisense oligonucleotides have shown that downregulation of AR expression is sufficient to slow tumor growth and to induce apoptosis. A new class of selective AR modulators (SARMs) targeting the hypersensitive pathway has been described recently. Nonsteroidal antiandrogens diarylthiohydantoins RD162 and MDV3100 bind to AR with greater affinity than the classical antiandrogen bicalutamide, reduce nuclear translocation of the AR (AR silencing), and impair both DNA binding to androgen response elements and recruitment of coactivators [53]. Both SARMs (RD162 and MDV3100) are orally available and induce tumor regression in mouse CRPCa models. A clinical phase I/II trial with MDV3100 enrolling 140 patients with CRPCa has shown sustained declines (by >50%) in serum PSA in 56% of patients, responses in soft tissue in 13 (22%) of 59 patients, stabilized bone disease in 61 (56%) of 109 patients, and conversion from unfavorable to favorable circulating tumor cell counts in 25 (49%) of the 51 patients. The median time to progression was 47 weeks for radiological progression [53]. It is conceivable that these SARMs (RD162 and MDV3100) targeting the hypersensitive pathway may also be effective to slow disease progression in patients at high risk to fail RT, and documented high level of AR expression in PCa tissue at the time of the initial diagnosis. Finally, natural agents, such as vitamin D3 and E, selenium, phytoestrogens, resveratrol, pomegranate fruit extract, and silymarin, have AR silencing activity documented in various PCa cell lines, [17, 21]. 

The hypersensitive pathway is further boosted by the increase of intratumoral testosterone synthesis. In fact, PCa uses cholesterol for intratumoral de novo testosterone synthesis, which is markedly increased in metastatic and CR disease [56]. Cholesterol lowering by statin use during high-dose RT for clinically localized prostate cancer has been reported to be associated with a significant improvement in freedom from biochemical failure, freedom from salvage androgen deprivation therapy, and relapse-free survival in high-risk patients, suggesting that statins have anticancer activity and possibly provide radiosensitization when used in conjunction with RT [54, 55]. The conversion of cholesterol to testosterone requires activity of the P450 aromatase enzyme (CYP17 gene) which can be blocked by abiraterone acetate [23, 56]. The use of abiraterone acetate may also prevent or delay RT failure in patients with high level of AR expression in their PCa tissue.

### 4.3. Targeting the Outlaw Pathway

Major growth factor receptors implicated in the development of RRPCa, including the EGFR family (erb1/EGFR, erb2/HER2, etc.) and VEGFR, require PI3K/AKT/mTOR downstream kinase pathways. This makes PI3K/AKT/mTOR signaling a promising target for radiosensitization [1, 59]. A number of PI3K inhibitors, including XL147 (Exelixis), BEZ235 (Novartis), GDC-0941 (Genentech)), and small-molecule AKT inhibitors (MK2206 (Merck, Inc.)) are currently in Phase 1 clinical trails (reviewed by Sarker et al., [59]). Preliminary results with mTOR inhibitors, such as rapamycin analogs (temsirolimus, everolimus) that inhibit the mTORC1 have been disappointing when these analogs have been administered as single agents in CRPC. Preliminary data on dual PI3K and mTOR inhibitors such as XL765 have recently been presented and encouragingly show no significant toxicity concerns and evidence of pharmacodynamic modulation [59]. The clinical success of agents targeting the PI3K/AKT/mTOR pathway could be maximized by prospectively identifying patients harboring molecular abnormalities in this pathway who may have a higher likelihood of responding, that is loss of PTEN demonstrated by FISH, high levels of AKT expression, and loss of p27 identified by immunohistochemistry in the patient's PCa tissue.

PI3K/AKT/mTOR downstream kinase pathways also regulate NF-**κ**B which, in turn, regulates AR expression and various other pathways implicated in cell survival, proliferation, invasion, angiogenesis, and metastasis (reviewed by Aggarwal et al., [34]). Numerous agents identified from natural sources can block the NF-**κ**B pathway, including curcumin, resveratrol, ursolic acid, capsaicin, silymarin, guggulsterone, and plumbagin [34]. In human clinical trials, curcumin has been shown to downregulate both the NF-**κ**B and Stat3 pathways [34].

Radiation can increase the expression of EGFR, which has been implicated in increasing the radiation resistance of cancer cells. A number of EGFR inhibitors including the monoclonal antibodies cetuximab (Erbitux) and panitumumab (Vectoibix) and small molecule tyrosine kinase inhibitors gefitinib (Iressa) and erlotinib (Tarceva) have shown potential in the treatment of several types of human cancers (reviewed by Karar and Maity, [33]). In a landmark randomized Phase III trial, treatment of locoregionally advanced head and neck cancer with concomitant high-dose radiotherapy plus cetuximab improves locoregional control and reduces mortality without increasing the common toxic effects associated with radiotherapy to the head and neck [70]. A recent Phase I/II trail of gefitinib combined with RT in patients with nonmetastatic PCa has shown promising clinical activity compared favorably with those of matched patients treated with radiation only at higher doses [57]. Erlotinib, another tyrosine kinase inhibitor targeting EGF-R, has shown moderate activity in chemotherapy-naïve CRPPCa, with 11% of patients meeting the criteria for partial response and 4% of patients demonstrating stable disease [58]. The VEGF pathway is targeted in current phase III trials enrolling patients with CRPCa with various agents, including bevacizumab, aflibercept, and sunitinib [23]. It is clear that tyrosine kinase inhibitors may be effective only in patients with tumors expressing pertinent growth factor receptors at significant level. With respect to the heterogeneous nature of PCa, determination of the erb1/EGFR, erb2/HER2, and VEGF-R status in PCa tissue would be the first step towards a better identification and selection of patients who will experience maximal benefit from each particular tyrosine kinase inhibitor (Figure 3(a)). 

Another multitarget approach to radiosensitization is provided by HSP-90 inhibitors. HSP-90 is a heat shock protein which stabilizes and prevents a number of relevant targets of CRPCa and RRPCa from degradation, including AR, HER-1, HER-2, and AKT. Inhibition of HSP-90 function by geldanamycin analogue results in degradation of these client molecules and impairs their biological functions [14, 17, 21, 22]. The geldanamycin analogues 17AAG and 17DMAG were shown to enhance the in vivo radiosensitivity of a prostate tumor xenograft model. The geldanamycin analogue 17-AAC was tested in a phase II trial in patients with CRPCa, but minimal clinical activity was recorded [23]. It is known that the HSP-90 inhibitor-induced tumor cell radiosensitization is closely related to the EGFR status (reviewed by Camphausen and Tofilon, [60]). In tumor cells expressing erbB3, erbB1 signaling was maintained in response to Hsp-90 inhibition and radiosensitization was not induced. With respect to the potential design of clinical protocols combining HSP-90 inhibitor and RT, the preclinical results suggest that patients with tumors which do not express erbB3 would be predicted to respond best to this combined modality and that the probability of HSP-90 inhibitor-induced radiosensitization may be enhanced by the combined use of erbB1 inhibitors [60].

### 4.4. Targeting the Bypass Pathway

Targeting the bcl-2 molecule-microtubule complex with docetaxel has let to the first shown survival benefit for patients with CRPCa [14]. Clinical studies have documented that bcl-2 positive PCa respond better to docetaxel than bcl-2 negative tumors [61]. Knowing that ADT and RT may induce and enhance bcl-2, determination of the bcl-2 status may be important in recurring PCa after RT (Figure 3(b)). In tumors expressing bcl-2 at significant levels, combination of ADT with low-dose docetaxel may be more effective than standard ADT alone (Figure 4(b)). In addition, bcl-2-positive PCa could be targeted by AT-101, which is a small-molecule inhibitor of multiple bcl-2 family members. Based on the promising results obtained in a single-agent Phase II trial, AT-101 is currently tested in a Phase II trial in patients with metastatic CRPCa treated with docetaxel [23]. When clinical activity is proven in this disease setting, AT-101 may become a promising agent for hormone- and radio-naïve PCa expressing bcl-2 at high level.

Clusterin is a cytoprotective chaperone that inhibits apoptosis and activates the PI3K/AKT kinase pathway implicated in the outlaw pathway [71]. In contrast to bcl-2, clusterin has no prognostic significance in hormone-naïve PCa, but is significantly upregulated under ADT [72]. OGX-011 is a clusterin silencing antisense oligonucleotide, which has shown promising clinical activity in patients with CRPCa treated with chemotherapy [23]. Clusterin silencing by OGX-011 may also be effective to slow disease progression in patients under ADT and RT. In fact, inhibition of clusterin by antisense oligonucleotides sensitizes LNCaP cells and PC-3 cells to RT [73]. 

Detection of significant NE differentiation in PCa (Figure 4(a)) has several implications for the patient's care [16, 74]. It is clear that radical prostatectomy eliminates the multidrug-resistant NE phenotype safer than RT or ADT. Knowing that NE PCa cells do not produce PSA, NE serum markers like CGA and neuron-specific enolase (NSE) may be more informative than PSA. It is well established that NE differentiation can be induced and enhanced by ADT. Clinical studies investigating CGA velocity under ADT indicate that castration therapy is significantly more effective in inducing the NE pathway than bicalutamide monotherapy [75]. Intermittent ADT significantly decreases CGA serum levels when compared to continuous ADT, indicating that intermittent ADT is safer than permanent ADT in PCa with significant NE differentiation [76]. It is noteworthy that NE differentiation is activated via interleukin 6 (IL-6) and PI3K/Akt/mTOR signaling, which could be targeted accordingly [77]. Another important target for this multidrug-resistant phenotype is the somatostatin receptor. In fact, NE PCa cells may express somatostatin receptors, which can be targeted by somatostatin analogues (Figure 4(b)). Objective clinical response and marked decrease of serum CGA have been reported in patients with CRPCa treated with lanreotide in combination with dexamethasone or ethinylestradiol [65–67]. Hence, evaluation of the somatostatin receptor status in PCa tissue may be important for targeting the NE phenotype with somatostatin analogues.

Increased COX-2 expression in PCa tissue is considered an independent risk factor for RT failure [47]. Preliminary clinical studies have shown that COX-2 inhibitors celecoxib and etoricoxib significantly extend PSA-free survival after radical prostatectomy, external radiation, and intermittent ADT [62–64]. It is noteworthy that the COX-2 status was not considered in these clinical studies. It is likely that the real benefit of COX-2 inhibitors in patients with COX-2 positive PCa is much higher than reported by these studies. Besides celecoxib and etoricoxib, there is a number of natural COX-2 inhibitors, including vitamin D, curcumin, resveratrol, green tea, and omega-3 fatty acids (fish oil) [21].

### 4.5. Targeting the Stem Cell Pathway

The cancer stem cell hypothesis predicts that cytotoxic agents (including radiation) may eliminate bulk tumor cells but spare rare cancer stem cells, which may account for the subsequent disease relapse after treatment. It has been shown that the PTEN/PI3K/AKT pathway is critical for the in vitro maintenance of CD133/CD44 PCa progenitors. In fact, inhibition of the PTEN/PI3K/AKT pathway by the dual PI3K/mTOR inhibitor NVP-BEZ235 leads to a decrease in the population of CD133/CD44 PCa progenitor cells in vivo. Moreover, the combination of NVP-BEZ235 and taxotere, which targets the bulk tumor, is significantly more effective in eradicating tumors in a prostate cancer xenograft model than monotherapy [68]. Another recent study reports that gamma-tocotrienols (gamma-T3) and vitamin-E constituents, downregulate the expression of PCa stem cell markers (CD133/CD44) in androgen-independent prostate cancer cell lines (PC-3 and DU145), suppress tumor initiation ability of these cells, and chemosensitizes PC-3 to taxotere [69]. These preliminary experimental data suggest that the efficacy of cytotoxic agents in PCa can be enhanced by targeting the stem cell pathway.

## 5. Conclusions

RT failure of prostate cancer results from a multifactorial and heterogeneous disease process including a wide variety of signaling and effector molecules that often depend on the genetic and epigenetic background. Most but not all of pathways and related tissue markers implicated in RT failure are also relevant for disease progression after surgery and androgen deprivation, and are detectable by routine immunohistochemistry in the patient's PCa tissue long before the clinical onset of ADT and RT failure. Among the various biomarkers tested by the RTOG, p16, Ki-67 combined with MDM2, COX-2, and PKA yielded the most robust data in predicting RT failure. With these markers it should be possible to identify patients at high risk to fail RT. The predictive power of other markers involved in RRPCa, including AR, EGFR family, VEGF-R, PI3K/Akt/mTOR, and NE differentiation, needs to be established. A number of tissue markers, including the hypersensitive AR, EGFR, VEGF-R, COX-2, and NF-**κ**B/Stat3 are promising to offer relevant targets for currently available drugs to enhance the efficacy of RT. With respect to the potential design of clinical protocols combining targeted therapy and RT, it is of paramount importance to ascertain that the target is present at significant levels in the patient's PCa tissue.

Strategies focusing more on early detection and prevention of RRPCa may be more effective to extend survival of PCa patients than attempts to improve the outcome of patients with clinically proven RT failure.

## Figures and Tables

**Table 1:** Selection of potential markers/targets and pathways implicated in the development of radioresistant prostate cancer, their significance in predicting radiation therapy (RT) failure, and potential therapeutic inventions/agents.

Marker/target	Pathway	Prediction of RT failure	Potential therapeutic inventions/agents
D2-40	Metastatic	‡ (7–11)	Pelvic radiation
OLN+	Metastatic	‡ (12)	Pelvic radiation
AR	Hypersensitive	‡ (28)	AR silencing, MDV3100 [53] Statins [54, 55], abiraterone [56]
PKA	Hypersensitive	‡ (4), (32)	AR silencing, PKA knockdown [31]
Ki-67/MDM2	Proliferation	‡ (48)	AR silencing, antisense-MDM2 [49]
p16	Proliferation	‡ (4)	AR silencing
erb1/EGFR (HER-1)	Outlaw	NYD	Gefitinib [15, 57] Erlotinib [58] PI3K/AKT/mTOR inhibitors [59] HSP-90 inhibitors [60]
erb2/HER2 (HER-2/neu)	Outlaw	NYD	Pertuzumab PI3K/AKT/mTOR inhibitors [59] HSP-90 inhibitors [60]
VEGF-R	Outlaw	NYD	Bevacizumab, aflibercept, sunitinib [23]
PI3K/AKT/mTOR	Outlaw	NYD	PI3K/AKT/mTOR inhibitors [59] HSP-90 inhibitors [60]
NF-*κ*B/Stat3	Bypass	± (4)	Curcumin [34]
Bcl-2	Bypass	± (4)	Docetaxel [61] AT-101 [23]
Clusterin	Bypass	±	OGX-001 [16]
COX-2	Bypass	‡ (47)	Celecoxib, etoricoxib [62–64]
CGA (somatostatin receptor)	Bypass	NYD	Lanreotide [65–67]
CD44/CD133	Stem cell	NYD	PI3K/mTOR inhibitor NVP-BEZ235 [68]
CD44/CD133	Stem cell	NYD	Gamma-tocotrienols [69]

OLN+: occult lymph node metastasis; AR: androgen receptor; PKA: protein kinase A; EGFR: epithelial growth factor receptor; VEGF-R: vascular endothelial growth factor receptor; PI3K/AKT/mTOR: phosphatidylinositol 3 kinase (PI3K)/AKT/mammalian target of rapamycin; NF-*κ*B/Stat3: transcription factors nuclear factor-kappa B (NF-*κ*B) and signal transducers and activators of transcription 3 (Stat3), COX-2: cyclooxygenase 2; CGA: chromogranin A;

‡: significant prediction of RT failure has been reported.

±: significant prediction of RT failure has not been reported.

NYD: not yet determined.
